# Association of Dietary Sodium-to-Potassium Ratio with Nutritional Composition, Micronutrient Intake, and Diet Quality in Brazilian Industrial Workers

**DOI:** 10.3390/nu17152483

**Published:** 2025-07-29

**Authors:** Anissa Melo Souza, Ingrid Wilza Leal Bezerra, Karina Gomes Torres, Gabriela Santana Pereira, Raiane Medeiros Costa, Antonio Gouveia Oliveira

**Affiliations:** 1 Postgraduate Program in Nutrition, Health Sciences Center, Federal University of Rio Grande do Norte, Natal 59078-900, RN, Brazil; anissa.souza.094@ufrn.edu.br (A.M.S.); karina.torres@ufrn.br (K.G.T.); gabriela.pereira.016@ufrn.edu.br (G.S.P.); raiane.costa.092@ufrn.edu.br (R.M.C.); antonio.gouveia@ufrn.br (A.G.O.); 2 Nutrition Department, Health Sciences Center, Federal University of Rio Grande do Norte, Natal 59078-900, RN, Brazil; 3 Pharmacy Department, Health Sciences Center, Federal University of Rio Grande do Norte, Natal 59078-900, RN, Brazil

**Keywords:** sodium-to-potassium ratio, 24 h dietary recall, diet quality, food groups, food serving sizes, micronutrient, manufacturing workers

## Abstract

Introduction: The sodium-to-potassium (Na:K) ratio in the diet is a critical biomarker for cardiovascular and metabolic health, yet global adherence to recommended levels remains poor. Objectives: The objective of this study was to identify dietary determinants of the dietary Na:K ratio and its associations with micronutrient intake and diet quality. Methods: An observational cross-sectional survey was conducted in a representative sample of manufacturing workers through a combined stratified proportional and two-stage probability sampling plan, with strata defined by company size and industrial sector from the state of Rio Grande do Norte, Brazil. Dietary intake was assessed using 24 h recalls via the Multiple Pass Method, with Na:K ratios calculated from quantified food composition data. Diet quality was assessed with the Diet Quality Index-International (DQI-I). Multiple linear regression was used to analyze associations of Na:K ratio with the study variables. Results: The survey was conducted in the state of Rio Grande do Norte, Brazil, in 921 randomly selected manufacturing workers. The sample mean age was 38.2 ± 10.7 years, 55.9% males, mean BMI 27.2 ± 4.80 kg/m^2^. The mean Na:K ratio was 1.97 ± 0.86, with only 0.54% of participants meeting the WHO recommended target (<0.57). Fast food (+3.29 mg/mg per serving, *p* < 0.001), rice, bread, and red meat significantly increased the ratio, while fruits (−0.16 mg/mg), dairy, white meat, and coffee were protective. Higher Na:K ratios were associated with lower intake of calcium, magnesium, phosphorus, and vitamins C, D, and E, as well as poorer diet quality (DQI-I score: −0.026 per 1 mg/mg increase, *p* < 0.001). Conclusions: These findings highlight the critical role of processed foods in elevating Na:K ratios and the potential for dietary modifications to improve both electrolyte balance and micronutrient adequacy in industrial workers. The study underscores the need for workplace interventions that simultaneously address sodium reduction, potassium enhancement, and overall diet quality improvement tailored to socioeconomic and cultural contexts, a triple approach not previously tested in intervention studies. Future studies should further investigate nutritional consequences of imbalanced Na:K intake.

## 1. Introduction

Modern dietary patterns, increasingly characterized by the overconsumption of ultra-processed foods, refined sugars, and unhealthy fats, have been strongly linked to adverse health outcomes [1]. Excessive intake of added sugars, particularly fructose and high-fructose corn syrup, contributes to metabolic dysregulation, promoting insulin resistance, non-alcoholic fatty liver disease (NAFLD), and type 2 diabetes [2]. Diets high in trans and saturated fats elevate low-density lipoprotein (LDL) cholesterol, accelerating atherosclerosis and cardiovascular disease [3]. Sodium consumption, primarily from processed and packaged foods, exacerbates hypertension [4], while insufficient dietary fiber disrupts gut microbiota diversity and impairs metabolic and immune function [5]. Emerging evidence underscores the deleterious effects of artificial additives [6], excessive alcohol consumption [7], and imbalanced omega-6 to omega-3 fatty acid ratios [8], which collectively foster chronic low-grade inflammation.

The sodium–potassium (Na:K) ratio in the human diet has emerged as a critical biomarker for assessing cardiovascular and metabolic health [9]. Sodium (Na) and potassium (K) are essential electrolytes that play interdependent roles in maintaining physiological homeostasis, including fluid balance, nerve transmission, and muscle function [10]. However, modern dietary patterns, characterized by excessive sodium intake and inadequate potassium consumption, have disrupted this balance, elevating the Na:K ratio to levels associated with increased risks of hypertension, cardiovascular disease (CVD), stroke, and chronic kidney disease (CKD). The World Health Organization (WHO) recommends a daily sodium intake of less than 2000 mg (5 g of salt) and a potassium intake of at least 3500 mg, translating to a Na:K ratio of approximately 0.57 [11]. Yet, global adherence to these guidelines remains poor, with populations often consuming ratios exceeding 1.5, driven by processed and ultra-processed foods and insufficient fruit and vegetable intake [12]. This deviation underscores the urgent need to understand dietary contributors to the Na:K ratio, particularly in occupational groups such as manufacturing workers, whose demanding schedules and limited access to healthy foods may exacerbate imbalances.

Sodium and potassium exert opposing effects on blood pressure regulation. Sodium promotes fluid retention and vascular resistance, while potassium enhances vasodilation and sodium excretion via renal mechanisms [13]. Epidemiological studies consistently demonstrate that a high Na:K ratio is a stronger predictor of hypertension than either nutrient alone [14]. For instance, the INTERSALT study, involving over 10,000 participants across 32 countries, revealed that populations with higher urinary Na:K ratios had significantly elevated systolic blood pressure [15]. Similarly, a meta-analysis by Ma et al. [16] found that every unit increase in the Na:K ratio was associated with a 24% greater risk of CVD mortality. These findings highlight the ratio’s clinical relevance, positioning it as a target for dietary interventions.

Despite clear guidelines, global sodium consumption averages 3950 mg/day, nearly double the recommended limit, while potassium intake lags at 2300 mg/day, well below the 3500 mg threshold [17]. This imbalance is particularly pronounced in industrialized nations, where processed and convenience foods, which are major sodium sources, dominate diets. Conversely, potassium-rich foods like fruits, vegetables, legumes, and dairy are under consumed, often due to cost, accessibility, or cultural preferences. The resultant elevated Na:K ratios are linked not only to cardiovascular outcomes but also to poorer diet quality. Diets high in sodium often correlate with low micronutrient density, as processed foods typically lack essential vitamins and minerals [18]. This duality suggests that addressing the Na:K ratio could simultaneously improve both electrolyte balance and overall nutritional adequacy.

Food groups contributing to the Na:K ratio have been explored in diverse populations. Processed meats, canned goods, bread, and savory snacks are primary sodium sources, while leafy greens, bananas, potatoes, and beans are key potassium contributors [19]. However, dietary patterns vary by socioeconomic and occupational factors. For example, shift workers, including manufacturing employees, often rely on ready-to-eat meals and vending machine snacks due to time constraints and limited cafeteria options, potentially exacerbating sodium intake [20]. A study by Leone et al. [21] identified that low-income populations, akin to many manufacturing workers, consume fewer fresh product items due to cost barriers, further skewing their Na:K ratios. Yet, few studies have specifically examined food group associations with the Na:K ratio in blue-collar workers, a gap this study aims to address.

Existing research on dietary determinants of the Na:K ratio offers mixed insights. A cross-sectional analysis of NHANES data found that 40% of sodium consumed came from the top 10 food categories, which included prepared foods with sodium added, such as deli meat sandwiches and pizza, and 43% of potassium consumed was from other 10 food categories, which included foods naturally low in sodium, like unflavored milk, fruit, vegetables, and prepared foods [22]. Conversely, a Japanese cohort study emphasized soups and sauces as major sodium sources, with potassium intake heavily dependent on vegetables and fruits, fish, and milk [23]. These discrepancies underscore the influence of cultural and regional dietary habits, suggesting that interventions must be context specific. Furthermore, while some studies correlate high Na:K ratios with lower intakes of calcium, magnesium, and vitamin C, nutrients abundant in fruits and vegetables, the relationship between Na:K ratios and micronutrient consumption remains underexplored. Understanding these associations could inform multifaceted dietary guidelines that target both electrolyte balance and micronutrient sufficiency.

The interaction between a high dietary Na:K ratio and poor diet quality contributes to adverse health outcomes through synergistic pathways involving electrolyte imbalance, chronic inflammation, and metabolic dysregulation. Elevated Na:K ratios impair renal sodium excretion and reduce potassium-mediated vasodilation, increasing blood pressure and arterial stiffness [24]. Poor diet quality further aggravates this by promoting oxidative stress and low-grade inflammation, key contributors to atherosclerosis [25]. Epidemiological studies demonstrate that populations with high Na:K ratios and low diet quality scores exhibit higher cardiovascular mortality, with sodium overload potentiating endothelial damage while potassium deficiency exacerbates cardiac strain [15,26]. Additionally, diets low in magnesium and calcium, common in poor-quality diets, worsen sodium retention, further increasing hypertension and chronic kidney disease risk [27]. High Na:K ratios correlate with insulin resistance and dyslipidemia, particularly when diets lack fiber and antioxidants [28]. Ultra-processed foods often displace whole foods rich in polyphenols and short-chain fatty acids, disrupting gut microbiota composition and increasing systemic inflammation [29]. This microbiota imbalance may further impair electrolyte regulation, creating a vicious cycle of metabolic dysfunction [30].

This study investigated the dietary determinants of the Na:K ratio among manufacturing workers and explored how this ratio relates to micronutrient consumption. Beyond a simple nutrient analysis, the study investigated the contribution of traditional food groups to the Na:K ratio in the diet, estimating the net contribution per serving. In addition, the study investigated whether there is an association of the Na:K ratio with diet quality. Manufacturing workers may be particularly at risk of electrolyte imbalance due to occupational stressors that may compromise dietary habits. Long work shifts, physical labor, and irregular mealtimes often lead to reliance on convenience foods, which are typically energy-dense but nutrient-poor [28]. Such patterns likely elevate Na:K ratios while depriving individuals of essential micronutrients. However, no study has concurrently examined food group contributions to the Na:K ratio and its relationship with micronutrient intake in this population. This dual focus is critical, as sodium reduction and potassium enhancement strategies must not inadvertently compromise overall dietary quality. While previous studies have established the association between dietary Na:K ratio and cardiovascular risk factors, several critical gaps remain unaddressed. By focusing specifically on Brazilian industrial workers, we provide the first comprehensive analysis of how workplace-related dietary patterns influence Na:K ratios. Unlike previous studies that examined Na:K ratios in isolation, we employ the Diet Quality Index-International (DQI-I) to simultaneously evaluate how electrolyte imbalance interacts with overall diet quality, which contributed to elucidating whether high Na:K ratios serve as a marker for broader nutritional deficiencies. Moving beyond nutrient-level analyses, we examined traditional food groups to identify specific groups (e.g., fast food, dairy, fruits) that most significantly impact Na:K ratios in this population.

By identifying specific food groups associated with elevated ratios, the study aims to inform targeted interventions tailored to this population’s unique challenges. Additionally, elucidating links between the Na:K ratio and micronutrients may reveal synergies for public health strategies that address both electrolyte imbalance and nutritional deficiencies. Given the paucity of data on occupational dietary patterns, this research contributes novel insights into how workplace environments shape nutritional health, offering a foundation for policies that promote accessible, balanced diets in industrial settings.

## 2. Materials and Methods

### 2.1. Survey Design

This was an observational, cross-sectional study based on a representative probability sample of manufacturing workers in the state of Rio Grande do Norte, Brazil. The manufacturing industry stands out as one of the most important segments for the Brazilian economy. According to data from the Ministry of Development, Industry, Trade, and Services, it represents 11.3% of Brazil’s GDP and, in 2024, recorded record exports of US$181.9 billion, justifying the interest of this study in this important market segment. The sampling strategy employed a stratified proportional two-stage design, with stratification based on company size (categorized as small for firms with fewer than 50 workers, medium for 50 to 500 workers, and large for more than 500 workers) and sector of activity (food and beverages, non-metallic minerals, and textiles), which represented the most prominent industries in the region.

Ethical approval was obtained from the Research Ethics Committee of the Hospital Universitário Onofre Lopes (CAAE 2.198.545/2017), and the study adhered to the principles of the Declaration of Helsinki. All participants provided written informed consent prior to data collection.

In the first sampling stage, manufacturing companies were selected from each stratum through simple random sampling, with the number of selected companies proportional to their representation in the state’s industrial landscape. The sampling frame with the identification of all companies in the state from each of the three defined sectors of activity was provided by the Federation of Industries of the State of Rio Grande do Norte (FIERN). In the second stage, a predetermined number of workers were randomly selected from each participating company using computer-generated random numbers, drawn from employee lists supplied by human resources departments.

Eligibility for the study required companies from the three specified sectors to provide written consent to participate. Workers included in the study were male and female workers aged 18 years or older with permanent employment contracts, while exclusions applied to pregnant women, temporary workers, interns, and employees in probationary periods.

### 2.2. Data Collection

Fieldwork was conducted through scheduled visits by the research team between Tuesdays and Saturdays. Randomly selected workers were approached during lunch breaks and invited to participate. Following consent, researchers collected biodemographic information, including age, sex, marital status, education level, and income. Anthropometric measurements included body mass index (BMI), calculated from weight and height measurements taken with a digital scale (Inner Scan, Tanita Corp., Tokyo, Japan) and a stadiometer (Sanny, São Bernardo do Campo, SP, Brazil), respectively, and waist circumference (WC), measured at the midpoint between the lower rib and iliac crest using a measuring tape (Cescorf, Porto Alegre, RS, Brazil). All procedures followed the guidelines of the Brazilian Food and Nutrition Surveillance System–SISVAN [31].

Dietary intake was assessed using the 24 h dietary recall (24HR) method, administered through the Multiple Pass Method (MPM) to enhance recall accuracy and completeness [32]. The 24HR is a widely employed method in nutritional epidemiology for estimating individual food and nutrient intakes. Participants were interviewed by trained personnel to recall all foods and beverages consumed during the previous 24 h period, including detailed descriptions, preparation methods, and quantities. The MPM structures the interview into distinct passes, starting with a quick list of consumed items, followed by detailed probing for forgotten foods (e.g., beverages, snacks, condiments), then a collection of detailed descriptions and portion sizes using household measures, and finally, a review of the entire recall for verification and additions.

Diet quality was assessed with the Diet Quality Index-International (DQI-I), a comprehensive tool designed to evaluate overall diet quality across diverse populations, facilitating cross-national comparisons [33]. Developed to address the limitations of region-specific indices, the DQI-I incorporates four key dimensions (variety, adequacy, moderation, and overall balance in nutrient intake). The index assesses dietary patterns by scoring food group diversity (food consumption across major categories), adequacy of nutrient-rich foods (such as fruits, vegetables, and whole grains), moderation of less desirable components (e.g., saturated fats, cholesterol, and empty calories), and macronutrient balance (evaluating the proportion of energy derived from carbohydrates, fats, and proteins). The scale total score ranges from 0 to 100 points, the components score from 0 to 20, 40, 30, and 10 points, respectively, with higher scores indicating better diet quality.

### 2.3. Nutritional Analysis

Food consumption data from the 24HR were quantified by weight and volume using standardized references, including direct weighing, photographic records, and technical manuals [34,35]. Nutritional analysis relied on Food Composition Tables [36,37,38] supplemented with information from food labels when necessary.

### 2.4. Nutritional Composition

Foods were classified according to the Brazilian Food Guide’s grouping system, which categorizes foods by biological, nutritional, and culinary characteristics. These groups were further subdivided into 44 subgroups to accommodate the diversity of reported foods and preparations. Mixed dishes were disaggregated into individual ingredients using Technical Preparation Sheets, while traditional Brazilian mixed foods, primarily composed of fresh or minimally processed ingredients, were classified according to their dominant component. For example, the dish *baião-de-dois*, typically prepared with a mixture of rice and beans, but containing a higher proportion of rice according to traditional culinary recipes, was classified in the rice group. Similarly, traditional corn-based dishes such as *canjica*, which includes culinary ingredients such as sugar and coconut milk, were classified in the corn group [39]. The serving size estimates established for each food group were based on specific bibliographic references, which use protocols related to food consumption, such as directly weighing foods to standardize their size (small, medium, large) and/or defining them in household measurements (one cup, one tablespoon, one glass, one ladle, one plate, one handful, etc.) [35,40].

### 2.5. Statistical Analysis

For sample size calculations, the sampling plan aimed to select 30 companies for the first stage and 30 workers sampled within each company, to achieve a total sample of 900 participants, according to the widely used 30-by-30 two-stage cluster sampled design method in population surveys.

Descriptive data are presented as mean ± standard deviation or n (%). Univariate and multivariate linear regression was used to investigate associations of Na:K ratio with food groups, micronutrient intake, and diet quality, with results presented as partial regression coefficients with 95% confidence intervals (CI). Statistical significance was assumed for *p* < 0.05. Stata 15 (Stata Corp., College Station, TX, USA) was used for statistical analysis.

## 3. Results

Between September 2017 and July 2018, the study enrolled 921 workers from 33 manufacturing companies across three key industrial sectors: food and beverages (14 companies), non-metallic minerals (6 companies), and textiles (13 companies). The participating companies represented a range of sizes, with 13 classified as small, 14 as medium, and 6 as large companies. Notably, the study achieved complete participation with no refusals and no missing or incomplete data, ensuring a robust dataset. Each worker interview was conducted efficiently, averaging less than 15 min in duration.

The participant demographic profile (Table 1) revealed a mean age of 38.2 ± 10.7 years, with a slight male predominance (55.9%). Most workers (62.7%) reported living with a partner, while the average income stood at 1.46 times Brazil’s minimum wage. Anthropometric measurements showed an average body mass index (BMI) of 27.2, with weight distribution categories indicating 34.4% of participants at normal weight, 39.0% overweight, and 25.4% meeting criteria for obesity.

The mean Na:K ratio was 1.97 ± 0.86, with only 0.54% (95% confidence interval (CI) 0.17–1.26%) below the WHO recommended value, and only 5.21% (95% CI 3.87–6.85%) with a Na:K ratio below 1.0.

Table 2 shows which food groups contribute to an increase or decrease in the daily Na:K ratio. Multivariate analysis shows those food groups independently associated with the Na:K ratio. The Na:K ratio decreases 0.16 mg/mg (*p* < 0.001) for each serving of fruit in the diet, 0.04 mg/mg for each serving of dairy products (*p* = 0.002), 0.13 for each serving of white meat (*p* < 0.001), and 0.21 for each serving of coffee (*p* < 0.001). Food groups in the diet that increase the Na:K ratio include rice (0.21, *p* < 0.001), corn (0.12, *p* < 0.001), bread (0.08, *p* < 0.001), pasta (0.06, *p* = 0.10), soup (0.11, *p* < 0.001), red meat (0.12, *p* < 0.001), vegetable oil (0.07, *p* = 0.047) and, above all, fast food (3.29, *p* < 0.001).

The Na:K ratio of diet is associated with the intake of micronutrients and vitamins. Table 3 shows the average increase or decrease in micronutrient intake per 1 mg/mg increase in Na:K ratio in the diet. Diets with increasing Na:K ratio are associated with decreased intake of several elements (calcium, magnesium, and phosphorus) and increased intake of iron. In the case of vitamins, the increased Na:K ratio in the diet is associated with decreased intake of niacin and vitamins C, D, and E.

Increased Na:K ratio in the diet is also associated with diet quality. Table 4 shows the difference in DQI-I score per 1 mg/mg increase in Na:K ratio in the diet. Total score decreases an average of 0.026 points per unit increase in Na:K ratio (*p* < 0.001), reflecting worse quality of diets with higher Na:K ratios. All DQI-I subscales are also negatively associated with the diet Na:K ratio, except for Overall Balance probably because this dimension primarily assesses macronutrient and fatty acid distribution, without accounting for micronutrient intake or electrolyte ratios.

The range of scores for the DQI-I and each component is shown in brackets, higher scores indicating better diet quality.

## 4. Discussion

This study provides critical insights into the dietary determinants of the sodium-to-potassium (Na:K) ratio and its broader nutritional implications among manufacturing workers, a population group of interest given the importance of the workforce to a country’s economy, with the distinct aspect of assessing the association of the Na:K ratio from a food perspective, based on the servings consumed, which allowed both the analysis of food consumption beyond nutrients and the association to diet quality using an international quality indicator.

### 4.1. Micronutrient Intake and the Relationship Between Electrolyte Balance and Diet Quality

The mean Na:K ratio observed in this population far exceeds the WHO-recommended threshold of 0.57, with less than 1% of participants meeting this guideline. This disparity mirrors global trends of excessive sodium intake and insufficient potassium consumption, particularly in low- and middle-income settings where processed foods dominate diets [11,17,26]. Notably, the Na:K ratio’s association with specific food groups underscores the dual burden of industrialization and occupational constraints on dietary habits. Fast food emerged as the strongest association to elevated Na:K ratios, a finding consistent with studies linking ultra-processed foods to poor electrolyte balance [22,29,30]. Conversely, fruits, dairy, white meat, and coffee were protective, aligning with evidence that potassium-rich foods mitigate sodium’s adverse effects [24]. These results highlight the need to contextualize dietary guidelines for blue-collar workers, whose reliance on convenient, energy-dense meals exacerbates nutritional imbalances [41].

The inverse relationship between the Na:K ratio and micronutrient adequacy further underscores the interconnectedness of dietary quality and electrolyte balance. Elevated ratios were associated with reduced intake of calcium, magnesium, and vitamins C, D, and E, which are nutrients abundant in fruits, vegetables, and minimally processed foods. This aligns with findings that sodium-dense diets often displace micronutrient-rich options, compounding cardiovascular risks [25]. The positive association with iron intake may reflect the prevalence of fortified processed foods or red meat consumption, which, while iron-rich, also contribute to sodium overload. Such trade-offs emphasize the complexity of addressing nutritional deficiencies in populations with limited access to fresh produce.

The observed decline in diet quality, as measured by the DQI-I scale, reinforces the Na:K ratio’s utility as a proxy for overall dietary patterns. An increase in the Na:K ratio is associated with a reduction in total DQI-I score, driven by poorer variety, adequacy, and moderation, a pattern consistent with studies linking high sodium intake to monotonous, nutrient-poor diets [42].

Numerous studies have investigated the association of high Na:K ratios with arterial hypertension and cardiovascular diseases, but there is limited information on the association of the Na:K ratio with food composition, micronutrient intake, and diet quality. A study based on the Irish Adult Nutrition Survey of a nationally representative sample of the adult population, with sodium and potassium urinary excretion measured with spot urine samples, also investigated foods associated with Na:K ratios with findings similar to those of the present study: fruits, vegetables, potatoes, breakfast cereals, milk, yogurt, and fresh meat contribute to a higher Na:K ratio, while the foods negatively associated with a lower Na:K were breads, cured and processed meats, and butter and fat spreads [43]. Another study conducted in healthy pregnant women also investigated the association of food groups to Na:K ratios, again with findings similar to those in this study, in which soups and sauces, cereals and cereal products, and fats and oils were associated with increased Na:K ratios, while fruit and non-alcoholic beverages were associated with decreased Na:K ratios [44]. However, we have no knowledge of studies investigating the association of Na:K ratios with micronutrient intake. In addition, although the Na:K ratio is being considered a proxy for diet quality because of its association with cardiovascular risk, a direct correlation with a validated measure of diet quality had not been previously studied. Furthermore, we are unaware of previous studies conducted on the population of manufacturing workers.

### 4.2. Methodological Considerations

For this research, we elected a single 24 h recall for estimating usual nutrient consumption because, although it may not fully capture habitual intake, it offers significant advantages when deployed in large-scale epidemiological surveys such as the present one. First, its administration is relatively quick and cost-effective per participant, compared to methods like food diaries, making it feasible for large samples. Second, it does not require participant literacy, thus reducing non-respondent bias. Third, it minimizes the long-term recall bias inherent in food frequency questionnaires by not relying on memory beyond the immediate past. On the other hand, although a single 24HR per individual captures only a snapshot of intake, which may not reflect habitual dietary patterns due to substantial day-to-day variation, particularly for episodically consumed foods and nutrients, the main consequence of such measurement error is to increase the variance of the distribution of daily intakes. While this may have significant impact on the estimation of individual-level intake, which is particularly worrisome in studies estimating the fraction of the population with usual intake above or below some standard [45], it may not be significant in studies estimating group-level usual intakes. This is supported by several studies in low-income countries, comparing weighted food records with a single 24 h recall, that have found no significant underreporting of intakes estimated at group level [46]. The effect in analyses involving regression is mostly an attenuation of the regression coefficients and the increase in their standard errors, which may lead to loss of statistical power and possibly to missing some significant associations.

Another methodological option was to estimate individual Na:K ratios from 24HR. While 24 h urinary excretion is considered the gold standard for estimating sodium and potassium intake, in large-scale surveys, spot urine measurements and 24HR are often used as alternatives due to practical constraints (such as the challenge of obtaining 24 h urine collections, the dependency on participant compliance, and complex logistics of storage, transportation and analysis), while improving participation rates and decreasing self-selection bias, although its limitations in accuracy are acknowledged.

Regarding the use of the DQI-I to evaluate diet quality, validation studies have demonstrated that the DQI-I correlates with biomarkers of nutritional status and health outcomes, supporting its construct validity [47]. Its adaptability allows for application in different cultural and dietary contexts while maintaining a standardized scoring system, enhancing comparability in epidemiological research [33]. However, like all diet quality indices, the DQI-I relies on accurate dietary intake data, typically obtained through 24HR or food frequency questionnaires, and may be influenced by measurement errors. Despite this limitation, its structured approach provides a useful framework for assessing dietary patterns in relation to public health goals, making it a valuable tool in nutritional surveillance and intervention studies.

### 4.3. Public Health Implications

The findings of this study carry significant public health implications. Manufacturing workers, often constrained by irregular schedules and limited meal options, face structural barriers to achieving dietary balance. Workplace interventions targeting sodium reduction, such as subsidized healthy meals, nutrition education, and restrictions on fast-food vendors, could mitigate these disparities [48]. Additionally, promoting potassium-rich foods like fruits and dairy through onsite cafeterias may counteract sodium’s effects. However, such strategies must account for socioeconomic realities, as cost and accessibility remain key determinants of food choice in this demographic group [49,50]. The regional specificity of dietary patterns, exemplified by the prominence of rice and corn as sodium contributors, further underscores the need for culturally tailored guidelines. For instance, traditional Brazilian dishes like feijoada, though nutrient-dense, may require modifications to reduce hidden sodium without compromising cultural acceptability [51].

### 4.4. Limitations and Strengths

This study has some limitations. Because it is based on a cross-sectional design, causal relationships between dietary patterns and nutritional outcomes cannot be inferred, but we did not make such inferences. Furthermore, dietary intake was self-reported, which may introduce recall bias, and focusing on the single Brazilian state limits generalizability to other occupational or cultural contexts. As previously discussed, the 24 h recall method has limitations in estimating habitual nutrient intake and sodium and potassium intake. However, to mitigate this limitation, we employed the Multiple Pass Method (MPM), a structured and validated approach known to improve the accuracy and completeness of dietary recall data.

Despite these limitations, the study presents several methodological strengths that increase the robustness and credibility of the results. The use of a probability sampling strategy ensured that the sample was representative of the working adult population across the state. The large sample size increases statistical power and the precision of the estimates. Moreover, data collection was conducted prospectively through in-person interviews conducted by trained nutritionists, minimizing potential measurement errors. It is important to highlight that the absence of non-response and the negligible risk of self-selection bias reinforce the internal validity of the study. Together, these aspects corroborate the reliability of our findings and reinforce their relevance for informing dietary interventions and public health policies focused on workforce nutrition.

## 5. Conclusions

This study investigated dietary Na:K ratio and its associations with diet composition, micronutrient intake, and overall diet quality among industrial workers. Less than 1% of workers met the WHO-recommended threshold for Na:K ratio. Key dietary contributors to elevated ratios included fast food, rice, bread, and red meat, while fruits, dairy, white meat, and coffee were protective. Higher Na:K ratios were associated with reduced intake of essential micronutrients such as calcium, magnesium, phosphorus, and vitamins C, D, and E, as well as poorer diet quality, as evidenced by lower Diet Quality Index-International scores. These findings underscore the dual burden of excessive sodium and inadequate potassium intake in this population, driven largely by processed and convenience foods. By demonstrating its inverse relationship with micronutrient adequacy and diet quality, this research reinforces the interconnectedness of electrolyte balance and nutritional health.

In conclusion, this study elucidates the multifactorial drivers of elevated Na:K ratios in manufacturing workers and their downstream effects on micronutrient intake and diet quality. By demonstrating its inverse relationship with micronutrient adequacy and diet quality, this research reinforces the interconnectedness of electrolyte balance and nutritional health.

Methodologically, estimating the change in Na:K ratio per serving of a food group (rather than per gram or kilocalorie) represents a meaningful methodological innovation that enhances the practical evaluation of dietary contributions to electrolyte balance. By linking Na:K ratios to standardized serving sizes, the findings become more interpretable and actionable for both public health messaging and individual dietary guidance. In addition, estimating the change in Na:K ratio per serving of a food group captures the combined effects of the multiple ingredients or processing steps in a single serving of a food item that collectively influences its sodium and potassium content. Another benefit of the approach is that it facilitates direct comparisons across food groups in a way that echoes dietary guidelines, which often recommend servings, simplifying the translation of study findings into interventions, such as promoting specific serving substitutions.

By identifying actionable dietary targets, such as reducing fast food and refined grain consumption while increasing fruits and dairy, these findings offer a roadmap for policies aimed at improving both electrolyte balance and nutritional adequacy in high-risk populations. Addressing these dual challenges is essential to curbing the growing burden of diet-related chronic diseases in industrial settings. In addition, to improve the unfavorable Na:K ratios observed among industrial workers, targeted nutritional interventions are needed at both the individual and environmental levels. Strategies could include reformulating commonly consumed processed and convenience foods to reduce sodium content and/or increase potassium density, along with workplace-based programs that promote the consumption of potassium-rich foods such as fruits, vegetables, legumes, and dairy products. In parallel, nutrition education campaigns tailored to this workforce could emphasize the benefits of replacing sodium-rich items (e.g., fast food, processed meats, white bread) with protective alternatives (e.g., whole foods, minimally processed options), using portion-based guidelines to align with the study’s methodological framework. These efforts could be further supported by institutional policies that improve the availability and accessibility of healthier options in workplace cafeterias and surrounding food environments, ultimately contributing to improved electrolyte balance and overall diet quality in this population. Future studies should further investigate nutritional consequences of imbalanced Na:K intake, particularly on micronutrient composition of diets, as well as on vascular, renal, hormonal, and metabolic functions, and on gut microbiota composition. Also, additional information is needed on the food groups with greater contribution to Na:K balance, while intervention trials could test the efficacy of workplace-based dietary programs.

## Figures and Tables

**Table 1:** Characteristics of the study population.

Variable	Values
Age, years	38.2 ± 10.7
Sex, male	515 (55.9%)
Married/Cohabiting	577 (62.7%)
Education > middle school	527 (57.3%)
Income, minimum wages	1.46 ± 1.61
Body Mass Index, kg/m^2^	27.2 ± 4.80
Waist Circumference, cm	89.7 ± 11.9
Underweight	12 (1.30%)
Normal weight	316 (34.4%)
Overweight	359 (39.0%)
Obesity I	178 (19.4%)
Obesity II	40 (4.35%)
Obesity III	15 (1.63%)
Na:K ratio (mg/mg)	1.97 ± 0.86
Na:K ratio > 0.6 mg/mg	5 (0.54%)
Na:K ratio > 1.0 mg/mg	48 (5.21%)

**Table 2:** Changes in Na:K ratios per one serving of each food group in the diet.

	Univariate Analysis				Multivariate Analysis			
Food Group	Coefficient	95% CI		*p*-Value	Coefficient	95% CI		*p*-Value
**Increase Na:K Ratio**								
Beans	0.050	0.018	0.082	0.002				
Rice	0.210	0.144	0.276	0.000	0.205	0.151	0.258	<0.001
Corn	0.092	0.043	0.140	0.000	0.115	0.076	0.154	<0.001
Bread	0.086	0.048	0.124	0.000	0.077	0.046	0.107	<0.001
Pasta	0.099	0.051	0.148	0.000	0.057	0.014	0.100	0.010
Soups	0.114	0.096	0.131	0.000	0.109	0.094	0.123	<0.001
Red meat	0.189	0.145	0.232	0.000	0.116	0.077	0.156	<0.001
Processed meat	0.162	0.082	0.243	0.000				
Fast food	3.332	1.644	5.020	0.000	3.294	1.943	4.645	<0.001
Vegetable oil	0.102	0.036	0.168	0.002	0.065	0.001	0.130	0.047
Salt	0.036	0.021	0.052	0.000				
**Decrease Na:K Ratio**								
Grains, integral	−0.153	−0.247	−0.058	0.002				
Tubercles	−0.105	−0.143	−0.067	0.000				
Fruits	−0.204	−0.254	−0.154	0.000	−0.158	−0.198	−0.117	<0.001
Dairy	−0.056	−0.090	−0.022	0.001	−0.042	−0.069	−0.015	0.002
White meat	−0.151	−0.206	−0.097	0.000	−0.129	−0.179	−0.079	<0.001
Coffee	−0.164	−0.222	−0.106	0.000	−0.208	−0.255	−0.161	<0.001
Vitamin supplements	−0.205	−0.375	−0.036	0.018				

**Table 3:** Association of Na:K ratio in the diet with intake of micronutrients.

Micronutrient	Coeficient	95% CI		*p*-Value
Calcium	−45.774	−76.059	−15.489	0.003
Copper	0.000	−0.209	0.210	0.99
Iron	1.813	1.151	2.476	<0.001
Magnesium	−13.451	−21.820	−5.081	0.002
Manganese	0.070	−0.274	0.413	0.69
Phosphorus	−58.971	−99.339	−18.603	0.004
Selenium	−3.014	−7.212	1.184	0.16
Zinc	1.069	−0.094	2.232	0.07
Vitamin A	−55.447	−267.550	156.656	0.61
Vitamin C	−28.809	−46.282	−11.336	0.001
Vitamin D	−0.379	−0.679	−0.078	0.013
Vitamin E	−0.691	−1.032	−0.350	<0.001
Cobalamin	−0.942	−2.144	0.260	0.13
Niacin	−1.262	−2.211	−0.313	0.009
Pyridoxine	0.011	−0.055	0.078	0.74
Riboflavin	0.047	−0.034	0.129	0.25
Thiamine	0.044	−0.010	0.098	0.11

**Table 4:** Change in DQI-I score and its components per 1 mg/mg increase in dietary Na:K ratio.

DQI-I Score	Coefficient	95% CI		*p*-Value
Total score (0–100)	−0.026	−0.032	−0.020	<0.001
Variety (0–20)	−0.046	−0.066	−0.026	<0.001
Adequacy (0–40)	−0.022	−0.034	−0.010	<0.001
Moderation (0–30)	−0.032	−0.040	−0.024	<0.001
Overall Balance (0–10)	0.018	−0.009	0.045	0.19

## Data Availability

The study data is available from the corresponding author upon reasonable request. Data are not publicly available due to ethical reasons as data requests will have to be reviewed by the Institutional Review Board.

## Abbreviations

The following abbreviations are used in this manuscript:

Na:K	Sodium-to-potassium
DQI-I	Diet Quality Index-International
BMI	Body Mass Index
24HR	24 h dietary recall
MPM	Multiple Pass Method
